# Comparison of Subcutaneous Versus Intramuscular Estradiol Administration for Feminizing Gender-Affirming Hormone Therapy

**DOI:** 10.3390/pharmacy14010013

**Published:** 2026-01-23

**Authors:** Abby C. Poage, Jordan M. Rowe, Mary Beth A. Dameron, Abigail M. Bavuso, Andrew J. Smith

**Affiliations:** 1 University Health Truman Medical Center, Kansas City, MO 64108, USA; abbycpoage@gmail.com (A.C.P.);; 2 University of Missouri-Kansas City School of Pharmacy, Kansas City, MO 64108, USA

**Keywords:** transgender, gender-affirming hormone therapy, transfeminine, estradiol, parenteral administration, subcutaneous administration

## Abstract

This single health system, retrospective cohort study compared subcutaneous (SC) versus intramuscular (IM) estradiol administration in 70 adult patients with a diagnosis of gender incongruence or gender dysphoria seen in an LGBTQ Specialty Clinic within a safety-net institution between October 2018 and December 2024. The primary endpoint was patients who reached therapeutic estradiol levels at 6 months. Secondary endpoints included the incidence of sub- and supra-therapeutic and actual estradiol levels at months 3, 6, 9, and 12 and patients who received pharmacist-led injection technique education. At 6 months, the proportion of patients achieving therapeutic estradiol levels did not differ between IM and SC administration. In exploratory analyses of continuous estradiol concentrations, IM administration was associated with higher measured estradiol levels.

## 1. Introduction

Gender-affirming hormone therapy (GAHT) is one of many potential strategies to address manifestations of gender dysphoria in the transgender and gender-diverse (TGD) population. This dysphoria can arise from the incongruence between an individual’s assigned gender and their experienced or expressed gender identity across multiple domains, including internal sense of self, social role, and desired physical characteristics [1,2,3]. Current guidelines recommend an estrogen with an androgen antagonist for feminizing GAHT, used with the goal of minimizing masculine characteristics and inducing feminine characteristics by increasing estradiol levels and suppressing serum testosterone. The primary estrogen used for feminizing GAHT is 17-beta-estradiol, commonly referred to as estradiol [4,5,6,7,8]. Estradiol is commonly commercially available in the United States in oral, transdermal, or injectable formulations, which may be administered as subcutaneous (SC) or intramuscular (IM) routes [4]. Conjugated estrogens and ethinyl estradiol are not recommended for GAHT due to erroneous serum estradiol levels and increased thromboembolic risk [4,5,6,9,10,11]. The Endocrine Society and World Professional Association for Transgender Health (WPATH) recommend that serum estradiol and testosterone levels are maintained at the levels for premenopausal females, which are 100–200 pg/mL and <50 pg/dL, respectively [4,6].

The most commonly prescribed injectable estradiol formulations in the United States are valerate and cypionate [4,5,6]. At the study institution, the primary formulation prescribed is estradiol valerate, and estradiol levels are monitored every 3 months following therapy initiation and dosage change. Once a standard dose has been established, monitoring extends to every 6 to 12 months if appropriate. Guidelines recommend a broad range of dosing for initiation and maintenance, suggesting an estradiol dose of 2–10 mg IM every week or 5–30 mg IM every 2 weeks [4,5,6]. However, recent evidence suggests that these dose ranges may be higher than necessary, often resulting in supratherapeutic estradiol levels [12,13]. A recent scoping review of estradiol dosing recommends starting injectable estradiol cypionate or valerate at ≤5 mg weekly, with titration to maintain levels within guideline-recommended ranges [12]. Recent data in transgender youth receiving estradiol indicate that serum hormone concentrations can be highly variable, and that both age and route of administration may influence pharmacokinetics and clinical outcomes [14]. Additionally, a 2025 publication illustrated that both route and dose significantly influence serum estradiol and testosterone concentrations, with IM estradiol associated with higher estradiol and lower testosterone compared to oral and transdermal routes, and a linear dose–response relationship observed for oral estradiol [13].

The current literature and guidelines generally consider both SC and IM administration of testosterone for masculinizing GAHT to result in clinically equivalent efficacy and serum testosterone levels [4,15,16]. In contrast, there is limited and conflicting literature regarding the differences in efficacy between SC and IM estradiol administration [12,13,14,17,18,19,20,21]. The purpose of this study was to compare SC vs. IM estradiol administration for feminizing GAHT to determine if route impacted time to therapeutic serum estradiol levels. To improve external validity and alignment for future systematic reviews, the authors’ study design sought to replicate a 2023 article comparing SC versus IM injectable estradiol [17], which is one of the most robust comparisons of IM and SC estradiol currently published. That study included 130 total patients (SC = 74; IM = 56) and found no differences in therapeutic estradiol levels between routes [17]. To our knowledge, this is the only study that specifically compares injectable routes of estradiol administration. Despite the availability of consensus guidelines, there remains substantial variability in clinical practice regarding estradiol dosing and route selection [4,5,6,12], and further research is needed to optimize regimens for individualized care. A 2025 article emphasized the importance of individualized dosing and shared decision-making, as well as the need for ongoing research to optimize regimens and monitoring strategies [18].

Common clinical practice at the study institution opts for a conservative approach when initiating patients on estradiol valerate, typically with dosing of 2–3 mg SC once weekly. Clinicians then typically target the serum estradiol and testosterone levels as measured with mid-injection laboratory assessment, as recommended by Endocrine Society and WPATH guidelines [4,6]. However, the guideline-recommended range for serum estradiol and testosterone levels may not be ideal for all patients. For example, TGD individuals desiring androgynous features may intentionally target sub-therapeutic estradiol and higher testosterone levels to align with individual goals for physical appearance [4,5,17].

In addition to a conservative initiation dosing strategy, the SC route is more frequently prescribed compared to the IM route for both masculinizing and feminizing GAHT. Clinicians prefer SC for most patients, as it allows use of a smaller needle with the potential for less associated pain and decreased scarring after long-term use [4]. Additionally, it is standard practice for patients in the clinic to receive pharmacist-led injection education upon initiation of injectable therapy. This education is individualized but consistently includes training on safe injection technique, sterile dose preparation, appropriately sized injection supplies, drug storage, expected effects and potential adverse effects, and visual demonstration using saline and an injection pad. The education concludes with the patient giving themselves their first injection with their home supply of injectable hormone for GAHT.

The purpose of this study was to compare SC vs. IM estradiol administration for feminizing GAHT to determine if route impacted time to therapeutic serum estradiol levels. Additional data in this area has the potential to advance GAHT practice and assist with future concrete recommendations for feminizing GAHT guidelines to align with current prescribing practices.

## 2. Materials and Methods

This retrospective single health system cohort study included individuals aged 18 and above diagnosed with gender incongruence, as defined by ICD-9 and ICD-10 codes. The study included patients seen at University Health LGBTQ Specialty Clinic in Kansas City, MO, USA between 1 October 2018, and 31 December 2024. Due to a transition in reporting software, data was unable to be collected prior to this time frame. Eligible patients had two or more serum estradiol levels available at least 3 months apart following initiation of SC or IM estradiol valerate. Patients were excluded if they had concurrent prescriptions for oral estradiol, estradiol patches, or estradiol cypionate, as well as if they were pregnant or incarcerated. Approval for the study was obtained from the institutional review board at the University Health Truman Medical Center.

Retrospective chart review and electronic health record (EHR) reporting functionalities were used for the extraction of demographic information, including age, race, and ethnicity, as well as clinical parameters, such as body mass index (BMI), antiandrogen use, additional estradiol prescriptions, smoking status, and weekly estradiol dose (see Table 1). The primary outcome was patients who reached therapeutic estradiol levels, defined as serum estradiol between 100 and 200 pg/mL, at 6 months. Secondary outcomes included incidence of sub- and supra-therapeutic and actual estradiol levels at months 3, 6, 9, and 12, testosterone levels at these same intervals, and patients who received pharmacist-led injection technique education.

Continuous data were analyzed using the Wilcoxon test, while categorical data were analyzed using the Chi-Squared and Fisher’s Exact tests. Statistical analyses were conducted using IBM^®^ SPSS Statistics^®^ Version 29.0.1.1. For the primary endpoint, logistic regression included route of administration as the sole predictor due to limited sample size and concerns regarding model stability. In exploratory linear regression analyses of continuous estradiol concentrations, estradiol dose was included as a covariate despite not being contemporaneous with the 6-month laboratory draw and was retained for exploratory purposes only to account for broad dosing differences rather than precise exposure at the time of measurement.

Because the primary endpoint (achievement of therapeutic estradiol levels at 6 months) is binary, logistic regression was used to evaluate the association between route of administration and therapeutic attainment. Multivariable regression analyses used listwise deletion and were restricted to patients with complete data for all variables included in each model. Adjustment for estradiol dose was not performed, as this reflected the most recently documented dose and was not reliably contemporaneous with the 6-month laboratory draw.

## 3. Results

A total of 70 patients were included in the study, with baseline characteristics described in Table 1. No differences were noted in baseline demographic information between the groups except for racial identity. Interpretation of differences between groups for specific racial identities is limited by small sample size. For example, while there were respectively 21.1% and 2% of patients in the IM and SC groups identifying as Black, the numerical difference was 4 versus 1 patient. Patients in the IM group trended older than those in the SC group (34.3 vs. 29.3 years, respectively), although this fell short of statistical significance (*p* > 0.05).

All included patients utilized once-weekly dosing for estradiol and were prescribed some form of antiandrogen. As patients switching from other forms of estradiol were included in the study (i.e., not only those newly starting feminizing GAHT), the range of baseline estradiol and testosterone levels varied widely. Additionally, not all participants had baseline labs drawn prior to IM or SC estradiol initiation. No other statistical differences in baseline characteristics were noted outside a difference between IM and SC in the overall characterization (therapeutic vs. not therapeutic) of baseline estradiol.

The distribution of estradiol levels at months 3, 6, 9, and 12 is detailed in Table 2 and Table 3. At each time interval, the range of patients in each group without estradiol laboratory data available ranged from 16.7% to as high as 73.7% in each group, limiting statistical power. BMI and testosterone levels also showed gaps in the available data at each time interval across patient groups. The average prescribed estradiol dose for the IM and SC groups was 3.81 mg and 3.32 mg, respectively. Logistic regression analysis evaluating the primary endpoint indicated that route of administration was not significantly associated with odds of achieving therapeutic estradiol levels at 6 months (odds ratio of 1.79, 95% CI 0.51–6.21, *p* = 0.36). Estradiol dose was included in this exploratory model as an imperfect proxy for treatment intensity and should be interpreted cautiously given the lack of standardized timing relative to laboratory assessment.

A separate, exploratory multiple linear regression was conducted evaluating predictors of continuous estradiol concentration at 6 months. This analysis included only patients with complete data for BMI, serum testosterone level, and estradiol dose (*n* = 43 patients), differing from the full cohort as no imputation or substitution was performed. Model degrees of freedom, effect estimates, and *p*-values were adjusted to reflect the complete-data sample. The overall regression model was statistically significant (F(4,38) = 4.30, *p* = 0.006). Given the limited sample size and variability in estradiol concentrations, this analysis should be considered exploratory.

Of the available data, SC users were less likely to have suppressed testosterone levels (serum testosterone less than 50 pg/dL) at 3 and 6 months compared to patients in the IM group (*p* < 0.01, *p* < 0.05 respectively). At the 9- and 12-month intervals, there were no statistical differences between the SC and IM groups in frequency of testosterone suppression (*p* > 0.05). Given the increasing proportion of missing laboratory data at later time points, particularly in the SC cohort, the reliability of results regarding estradiol levels at 9 and 12 months are limited.

Patients were assessed by chart review to confirm if they received injection education upon injectable estradiol initiation. A total of 13 of the 19 IM group patients received injection education, whereas 49 of the 51 SC group patients received injection education (*p* = 0.004).

## 4. Discussion

Although composed of a limited sample size, our study contributes additional data to the body of literature regarding routes of injectable estradiol for feminizing GAHT. The results of the multiple linear regression for the primary outcome suggest that even when controlling for dose, BMI, and serum testosterone, individuals receiving SC estradiol had significantly lower serum estradiol levels than those receiving IM injections. This may reflect differences in absorption, metabolism, or injection technique and supports further research on pharmacokinetics, pharmacodynamics, and dose adjustment strategies for SC and IM administration of estradiol in the TGD population. Estradiol dose showed a trend toward significance (B = 51.86, *p* = 0.075), suggesting a positive association with serum estradiol. Neither BMI (*p* = 0.252) nor testosterone level (*p* = 0.392) were significant predictors. These findings contribute to the literature suggesting that while both IM and SC estradiol can be used safely and efficaciously for feminizing GAHT, there may be clinically relevant differences in the dosing strategy required to reach the same serum estradiol and testosterone levels.

Additionally, the study results contribute to the growing body of literature indicating that starting estradiol doses for injectable therapy should be much lower than originally recommended in guidelines [4,5,6,12,13]. Although there was a wide range of serum estradiol levels amongst participants over the study period, most were prescribed 2 to 4 mg estradiol weekly, with many achieving therapeutic goal-range levels, or even supratherapeutic levels, with these doses. This real-world dosing data differs from the dosing range currently recommended by guidelines of 2–10 mg weekly and may support updates to future versions of GAHT guidelines regarding appropriate starting and typical maintenance doses for injectable estradiol.

Despite using a practice model targeting guideline-directed estradiol and testosterone levels [4,6], data showed instances where estradiol concentrations demonstrated substantial inter-individual variability across follow-up intervals, including occasional high values at earlier time points. Although such variability is expected in clinical practice, particularly with injectable formulations, it underscores the challenges of modeling estradiol levels in small retrospective cohorts and reinforces the exploratory nature of continuous-level analyses.

Chart review determined multiple factors contributing to this variability, including some discrepancies between prescribed dose and the dose patients administered. Struggles with medication adherence due to factors such as inconsistent insurance coverage and injection supply availability, affordability issues, adverse effects, and drug shortages were also noted. The influence of such factors aligns with other literature published regarding injection practices for GAHT [22] and warrants further prospective study. Additionally, the retrospective nature and format of data reporting made it challenging to identify if estradiol and testosterone levels were intentionally not within the target range due to individualized patient goals, such as desire for androgenous physical features, or if estradiol and testosterone levels had not yet reached optimal levels and required further therapy adjustment. Additional studies within the non-binary and gender-diverse population on estradiol feminizing therapy are also needed to identify optimal dosing strategies.

Due to the relatively small sample size, occurrence difference between groups, and outliers in estradiol serum levels in both the IM and SC groups, no definitive conclusions can be drawn about the impact of pharmacist-led self-injection education on improved efficacy or safety of either estradiol route. Although this study was not designed to describe or compare injection practices, anecdotally the authors found that patients who did receive pharmacist-led education upon injection initiation experienced less suboptimal injection practices related to dose, route, appropriate injection supplies, or other components related to safe self-injections. Further studies regarding injection education are needed and could further elucidate the role injection practices and experiences play in safety, time to therapeutic estradiol levels, clinical efficacy, and overall patient satisfaction with GAHT, as well as the role education may play in impacting such factors. Specifically, integration of pharmacists at both the clinic and community pharmacy levels of care is ideally poised to contribute to improved safety and efficacy with GAHT injections [22,23]. Although no validated instrument to assess patients’ self-injection experiences currently exists, pharmacists are trained to provide holistic education for medications, including medications administered via self-injection, and to identify safe versus suboptimal injection practices [23]. With their level of medication expertise, training on safe medication administration, and accessibility to the community [22,23,24], a more standardized approach by pharmacists regarding self-injection assessment and education for patients receiving GAHT could contribute to improved, person-centered care.

Our study has several limitations. Due to its retrospective nature and reliance on chart review, investigators were unable to conclusively determine some details initially planned to be assessed, such as gonadectomy status, venous thromboembolism incidence, and total duration of estradiol therapy inclusive of all formulations and routes. It was also unable to be determined if serum hormone values reflected the guideline-recommended mid-week laboratory draw (i.e., the half-way point between injections). This limitation is particularly important when comparing IM and SC administration, as the two routes may differ in absorption kinetics and concentration–time profiles. As a result, observed differences in measured serum estradiol concentrations may reflect variation in sampling timing rather than true route-specific pharmacokinetic differences, potentially modifying interpretation and subsequent clinical decision-making for dose adjustment. These findings should therefore be interpreted cautiously, and prospective studies with standardized laboratory timing are needed to clarify route-related pharmacokinetics.

An additional limitation results from a substantial portion of missing estradiol laboratory values at later intervals, particularly at 9 and 12 months, and among patients receiving SC estradiol. These missing data may introduce censoring or attrition bias, limiting the reliability of conclusions at 9 and 12 months. As this was a retrospective study relying on routine clinical follow-up, laboratory assessments and follow-up visits were not consistently obtained at each 3-month interval, and missing data are not statistically random. Accordingly, analyses beyond 6 months should be interpreted cautiously and are best considered exploratory.

Additionally, the inconsistency in the availability of EHR data limit the ability to utilize some statistical assessments, such as more detailed multivariate analyses. The relatively small IM cohort and imbalance in sample size between groups also limit statistical power and increase the risk of model instability and overfitting in multivariate analyses. Furthermore, the substantial variability in estradiol concentrations raises the possibility that individual outlier observations may disproportionately influence regression estimates. Therefore, regression findings should be interpreted as exploratory and hypothesis-generating rather than confirmatory.

Gaps in data for key variables were seen across all patient groups and at every time interval. Although this limits overall interpretation and validity, investigators posit that this is a realistic representation of patient data from clinics providing GAHT. TGD patients are more likely to experience barriers to care compared to the general population, and these barriers often lead to difficulties with medication access and consistent follow-up for laboratory and clinical assessment. Such barriers combined with baseline higher rates of negative social determinants of health are known to be influential factors in TGD care [4,22,23,25,26]. The study site is a safety-net institution that both provides care to an urban population but also is utilized by many patients from surrounding rural areas of the Midwest seeking a medical home prioritizing LGBTQ-affirming care. Therefore, investigators feel that while more complete and robust study data would be ideal, the study is still a valuable addition to the current literature on the subject, representing real-world patients experiencing realistic barriers to care. These findings also highlight the challenges of modeling estradiol concentrations in small, retrospective cohorts and underscore the need for larger, prospective studies with standardized laboratory timing to confirm route-specific pharmacokinetic and safety differences.

## 5. Conclusions

The purpose of this study was to compare SC vs. IM estradiol administration for feminizing GAHT to determine if route impacted clinical effect and time to therapeutic serum estradiol levels. In this retrospective cohort, study results demonstrate that achievement of therapeutic estradiol levels at 6 months did not differ by route of administration. Exploratory analyses suggest that IM estradiol was associated with higher measured serum estradiol concentrations, highlighting potential pharmacokinetic differences between routes and the need for individualized dosing strategies. These findings further contribute to the limited and conflicting literature regarding the differences in efficacy and pharmacokinetics between SC and IM estradiol administration, emphasizing the need for larger, prospective studies with standardized dosing strategies and laboratory timing to elucidate best clinical practices [12,13,14,17,18,19,20,21].

Results also affirmed recent literature demonstrating that lower doses of injectable estradiol than previously recommended are likely sufficient to achieve goal therapeutic levels in most patients. Further studies on this topic are needed to better describe current real-world prescribing and administration practices and specify optimum dosing strategies for injectable estradiol for feminizing GAHT. Finally, the study also highlighted the need for more systematic and robust assessment of and potential role of education for injection practices in individuals receiving injectable GAHT to appropriately optimize medication therapy. Pharmacists, who are optimally trained as medication experts and positioned as one of the most easily accessible health care providers within the United States, could be a key member of the interprofessional team to step into this gap in care.

## Figures and Tables

**Table 1:** Baseline characteristics.

	IM Estradiol *n* = 19	SC Estradiol *n* = 51	*p* Value
Mean age, y (SD)	34.3 (11.3)	29.3 (8.4)	0.052
Race			0.012
White (*n*, (%) ^1^)	12 (63.2%)	45 (88.2%)	
Black (*n*, (%))	4 (21.1%)	1 (2.0%)	
Other (*n*, (%))	1 (5.3%)	5 (9.8%)	
American Indian or Alaskan Native (*n*, (%))	1 (5.3%)	0 (0%)	
Native Hawaiian or Pacific Islander (*n*, (%))	1 (5.3%)	0 (0%)	
Ethnicity			0.195
Hispanic, Latino, or Spanish Origin (*n*, (%))	1 (5.3%)	0 (0%)	
Not Hispanic, Latino, or Spanish Origin (*n*, (%))	17 (89.5%)	45 (88.2%)	
Unavailable (*n*, (%))	1 (5.3%)	6 (11.8%)	
Gender identity			0.472
Female (*n*, (%))	18 (94.7%)	50 (98.0%)	
Non-binary (*n*, (%))	1 (5.3%)	1 (2.0%)	
Baseline body mass index, kg/m^2^ (SD)	25.9 (7.0)	26.1 (12.6)	0.947
Anti-androgen use			0.704
Spironolactone	18 (94.7%)	48 (94.1%)	
Finasteride	1 (5.3%)	3 (5.9%)	
Previous oral estradiol use (*n*, (%))	14 (73.7%)	42 (82.4%)	0.505
Previous estradiol patch use (*n*, (%))	1 (5.3%)	10 (19.6%)	0.267
Previous estradiol cypionate use (*n*, (%))	3 (15.8%)	1 (2.0%)	0.058
Baseline serum estradiol			0.006
No level available (*n*, (%))	7 (36.8%)	8 (15.7%)	
Therapeutic (*n*, (%))	4 (21.1%)	12 (23.5%)	
Sub- or supra-therapeutic (*n*, (%))	8 (42.1%)	31 (60.8%)	
Sub-therapeutic	4 (21.1%)	29 (56.9%)	
Supra-therapeutic	4 (21.1%)	2 (3.9%)	
Mean baseline serum estradiol level, pg/mL (SD) ^2^	175.9 (122.2)	145.0 (281.1)	0.594
Mean baseline serum testosterone level, pg/dL (SD) ^3^	159.9 (203.8)	171.4 (159.8)	0.956

^1^ Please note that percentages may not add to exactly 100 due to rounding; ^2^ of those with baseline serum estradiol levels; ^3^ of those with baseline serum testosterone levels.

**Table 2:** Primary outcome and key secondary outcomes at 3 and 6 months.

	IM Estradiol *n* = 19 ^1^	SC Estradiol *n* = 51 ^1^	*p* Value
Primary Outcome:			
Therapeutic serum estradiol at month 6 (*n*, (%))	6 (31.6%)	15 (29.4%)	0.34 ^2^
Secondary Outcomes:			
Serum estradiol at 3 months			0.047
No level available (*n*, (%)) ^3^	14 (73.7%)	20 (39.2%)	
Therapeutic (*n*, (%))	3 (15.8%)	13 (25.5%)	
Sub-therapeutic (*n*, (%))	0 (0%)	10 (19.6%)	
Supra-therapeutic (*n*, (%))	2 (10.5%)	8 (15.7%)	
Mean serum estradiol level at 3 months, pg/mL (SD)	475.6 (656.6)	156.2 (122.9)	0.159
Serum estradiol at 6 months			0.041
No level available (*n*, (%))	3 (15.8%)	22 (43.1%)	
Therapeutic (*n*, (%))	6 (31.6%)	15 (29.4%)	
Sub-therapeutic (*n*, (%))	2 (10.5%)	7 (13.7%)	
Supra-therapeutic (*n*, (%))	8 (42.1%)	7 (13.7%)	
Mean serum estradiol level at 6 months, pg/mL ± SD	294.3 (279.3)	131.2 (115.8)	0.037
Final weekly estradiol dose, mg (SD) ^4^	3.8 (1.3)	3.3 (0.7)	0.127

^1^ Not all patients had lab values at each assessment interval; values for each outcome represent the available data; ^2^ represents unadjusted *p* value, not representative of impact on controlling variables; ^3^ please note that percentages may not add to exactly 100 due to rounding; ^4^ represents last documented dose for latest interval with available data.

**Table 3:** Exploratory secondary outcomes at 9 and 12 months.

	IM Estradiol *n* = 19 ^1^	SC Estradiol *n* = 51 ^1^	*p* Value
Serum estradiol at 9 months			0.007
No level available (*n*, (%)) ^2^	7 (36.8%)	31 (60.8%)	
Therapeutic (*n*, (%))	2 (10.5%)	9 (17.6%)	
Sub-therapeutic (*n*, (%))	1 (5.3%)	6 (11.8%)	
Supra-therapeutic (*n*, (%))	9 (47.4%)	5 (9.8%)	
Mean serum estradiol level at 9 months, pg/mL ± SD	454.7 (562.4)	89.4 (97.9)	0.046
Serum estradiol at 12 months			0.067
No level available (*n*, (%))	6 (31.6%)	29 (56.9%)	
Therapeutic (*n*, (%))	5 (26.3%)	10 (19.6%)	
Sub-therapeutic (*n*, (%))	5 (26.3%)	3 (5.9%)	
Supra-therapeutic (*n*, (%))	3 (15.8%)	9 (17.6%)	
Mean serum estradiol level at 12 months, pg/mL (SD)	195.5 (264.1)	123.8 (164.6)	0.246

^1^ Not all patients had lab values at each assessment interval; values for each outcome represent the available data; ^2^ please note that percentages may not add to exactly 100 due to rounding.

## Data Availability

The data presented in this study are available on request from the corresponding author due to the small sample size and potential risk for patient de-identification for a patient population (transgender and gender-diverse individuals) who are at high risk for discrimination and other harms.
